# Evaluating Experimental Bias and Completeness in Comparative Phosphoproteomics Analysis

**DOI:** 10.1371/journal.pone.0023276

**Published:** 2011-08-10

**Authors:** Jos Boekhorst, Paul J. Boersema, Bastiaan B. J. Tops, Bas van Breukelen, Albert J. R. Heck, Berend Snel

**Affiliations:** 1 Theoretical Biology and Bioinformatics, Department of Biology, Faculty of Science, Utrecht University, Utrecht, The Netherlands; 2 Biomolecular Mass Spectrometry and Proteomics Group, Bijvoet Center for Biomolecular Research and Utrecht Institute for Pharmaceutical Sciences, Utrecht University, Utrecht, The Netherlands; 3 Netherlands Consortium for Systems Biology (NCSB), Amsterdam, The Netherlands; 4 Centre for Biosystems Genomics, Wageningen, The Netherlands; 5 Department of Proteomics and Signal Transduction, Max Planck Institute of Biochemistry, Martinsried, Germany; National Institutes of Health, United States of America

## Abstract

Unraveling the functional dynamics of phosphorylation networks is a crucial step in understanding the way in which biological networks form a living cell. Recently there has been an enormous increase in the number of measured phosphorylation events. Nevertheless, comparative and integrative analysis of phosphoproteomes is confounded by incomplete coverage and biases introduced by different experimental workflows. As a result, we cannot differentiate whether phosphosites indentified in only one or two samples are the result of condition or species specific phosphorylation, or reflect missing data. Here, we evaluate the impact of incomplete phosphoproteomics datasets on comparative analysis, and we present bioinformatics strategies to quantify the impact of different experimental workflows on measured phosphoproteomes. We show that plotting the saturation in observed phosphosites in replicates provides a reproducible picture of the extent of a particular phosphoproteome. Still, we are still far away from a complete picture of the total human phosphoproteome. The impact of different experimental techniques on the similarity between phosphoproteomes can be estimated by comparing datasets from different experimental pipelines to a common reference. Our results show that comparative analysis is most powerful when datasets have been generated using the same experimental workflow. We show this experimentally by measuring the tyrosine phosphoproteome from *Caenorhabditis elegans* and comparing it to the tyrosine phosphoproteome of HeLa cells, resulting in an overlap of about 4%. This overlap between very different organisms represents a three-fold increase when compared to dataset of older studies, wherein different workflows were used. The strategies we suggest enable an estimation of the impact of differences in experimental workflows on the overlap between datasets. This will allow us to perform comparative analyses not only on datasets specifically generated for this purpose, but also to extract insights through comparative analysis of the ever-increasing wealth of publically available phosphorylation data.

## Introduction

Phosphorylation of proteins is a key process in the complex interaction networks forming a living cell [1]. Many forms of adaptation in response to changing environmental conditions are regulated through phosphorylation, and changes in phosphorylation networks are likely to be an important source of phenotypic diversity [2], [3]. Novel experimental techniques have resulted in a huge increase in the amount of phosphorylation data available [4], providing the basis for the analysis of phosphorylation as a systems level property.

Analysis of phosphoproteomes from 11 yeast species (three measured and eight predicted) has provided putative evolutionary histories for the kinase regulation of protein complexes, and showed that mutations that result in changes in kinase–substrate interactions are an important source of phenotypic diversity [2]. Comparative phosphoproteomics has revealed significant evolutionary and functional signals in the overlap between phosphoproteomes [5], and the set of proteins with phosphorylation sites identified in different species of eukaryotes is enriched for disease-associated genes [6].

Although the evolutionary signal as well as the functional signal is significant, in absolute terms the overlap between phosphoproteomes is small [5]. This small overlap is not only the result of real differential phosphorylation (i.e. phosphosites present in one species and not in another, or sites phosphorylated under one condition but under another) but also of limitations of experimental techniques. The same factors also impact evolutionary analysis and function prediction of specific phosphosites on the basis of comparative analysis: differential phosphorylation is only meaningful when it represents a real difference in phosphorylation status, and is not the result of missing data caused by biases in experimental workflows or the incomprehensive nature of the datasets used.

**Figure 1:**
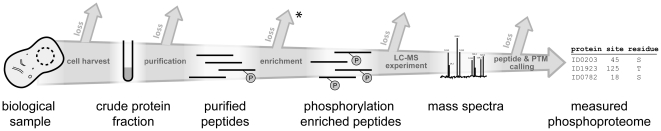
Outline of a high-throughput mass spectrometry based phosphoproteomics workflow. The horizontal arrow represents the number of phosphosites under analysis; the smaller arrows represent phosphosites lost at specific steps of the workflow. The arrow marked with a * (phosphosites not measured because they are lost in the enrichment phase) is discussed in more detail in the main text.

In the generally used high-throughput (HTP) mass spectrometry (MS) workflows, phosphorylation sites are potentially lost at all intermediary steps of such an experiment going from a biological sample to a list of putative phosphopeptides (fig 1): some phosphoproteins are relatively difficult to purify, kinase and phosphatase activity may still be ongoing to different degrees in the lysates, enrichment for phosphopeptides favors certain amino acid compositions in the phosphopeptides, etc. [4]. Targeted high-throughput MS approaches like multiple reaction monitoring (MRM) [7] can partly remove problems introduced by the incomprehensive nature of conventional HTP MS-based experiments. However, drawbacks are that a relatively small number of sites can be monitored [4], and an MRM experiment does not allow the identification of novel phosphosites, hence we will focus on conventional HTP experiments in our study. We analyze the impact of differences in experimental workflows on the observed overlap between phosphoproteomes. We study both the overlap between experiments investigating the same biological system using different experimental techniques, as well as the overlap between phosphoproteomes from different species.

An intuitive way to appreciate the amount of overlap between phosphoproteomics experiments would be to relate the number of phosphosites identified in both experiments to the total number of phosphosites in the complete phosphoproteome. Such a “complete human phosphoproteome” is an inventory of all amino acid residues in the human proteome that are phosphorylated under one or more conditions. However because of the incomprehensive nature of experimental workflows and conditions this total size is difficult to infer. Nevertheless we here implicitly obtain an estimate of the size of this complete human phosphoproteome. We collect data from a wide range of experiments, to estimate the relative completeness of different phosphoproteomes or sub-phosphoproteomes (e.g. a functional network related phosphoproteome, the phosphotyrosine proteome, or the phosphoproteome obtainable with a single workflow). Subsequently we quantify the impact of enrichment strategies by comparing the overlap between experiments that analyze similar biological systems using different enrichment strategies to a common reference experiment. We conclude our analysis by applying our insights from intra species comparative analysis to the analysis of phosphoproteomes from different species. Earlier analysis [5] showed a significant but very small overlap between phosphoproteomes from different species. We test how much we can improve this overlap by quantifying the overlap between the tyrosine phosphoproteome of human HeLa cells [8] and the tyrosine phosphoproteome of *C. elegans*, acquired using identical experimental workflows in a single laboratory.

## Results and Discussion

### The ever-expanding phosphoproteomes

Recent years have seen a rapid increase in high-throughput phosphoproteomics techniques and strategies [9], [10]. Yet, even using the most-advanced approaches, a single LC-MS run does not reveal all phosphosites in a biological sample: repeating the experiment always reveals novel sites, often up to 50% in a duplicate [11]. We here exploit this saturation effect to estimate the completeness of phosphoproteomics datasets by plotting how many unique/novel phosphosites are found relative to the number of replicate experiments or the total number of phosphosites that have been measured. We initially compared saturation in two different cases.

**Figure 2:**
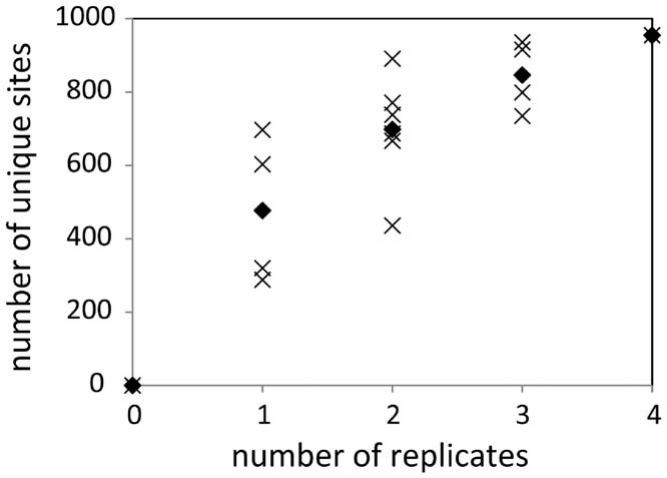
Saturation of the number of observed phosphosites in four replicates of an anti-Yp experiment. Phosphorylation data was taken from [12]. Labels on the horizontal axis represent the number of technical replicates, the vertical axis the number of unique phosphosites observed in these replicates. The crosses represent the different ways the relevant number of replicates can be picked from the total of four (there are four ways to pick one replicate, six ways to pick two replicates, etc). Diamonds are the average number of unique sites for that number of replicates. Generation and interpretation of the figure is described in more detail in the main text.

First, we investigated four replicates of an experiment measuring tyrosine phosphorylation in human stem cells (hESC) [12] (955 different sites). We measure saturation by plotting the number of combined replicates against the number of unique phosphosites observed (fig 2). A steep increase in the number of unique sites reflects that an additional replicate provides many sites that had not been observed before. An increase of zero would indicate the experiment has reached saturation, reflecting reproducibility of the experimental workflow: all sites phosphorylated under the specific conditions studied that can be detected within the limitations of the experimental set-up have been seen at least once. Replicate experiments of tyrosine phosphorylation in hESC cells are highly saturated. Only 15% of the sites found in the final repeat of the experiment had not yet been identified in earlier runs. This saturation could indicate we are close to fully sampling the tyrosine phosphoproteome of hESC under the specific conditions of the experiment, but it could also in part reflect the selective nature of the anti-phosphotyrosine (anti-Yp) enrichment strategy used in the experiment (i.e. replicates consistently sample the same subpopulation).

We assessed this data versus a combination of all human phosphorylation sites reported by HPRD [13] and the phospho.ELM [14] metadatabases (17693 different sites). For this comparison we cannot use the procedure described above, as this combined dataset contains phosphosites from a wide range of experiments, many of which are low-throughput. In addition, the total number of sites in the combined dataset is an order of magnitude higher than the number of sites in the HeLa experiment, hampering direct comparison. Instead, saturation is determined by counting the number of unique phosphosites relative to the total number of phosphosites measured. In short, this is done by pooling phosphorylation sites, adding a copy of a site to this pool for every observation of this specific site. For the hESC experiment, the number of copies of a site in the pool is equal to the number of replicates in which the site was observed. For the complete human phosphoproteome it is equal to the number of publications in which the site is reported (both phospho.ELM and HPRD provide a list of Pubmed identifiers for every site). We then start drawing random phosphorylation sites from this pool, and plot the fraction of draws relative to the total number of sites in the pool against the fraction of unique sites observed so far. The resulting curve represents saturation: when a system is nearly completely sampled, additional draws should reveal hardly any novel phosphorylation sites. Note that we randomly draw sites; the data presented in the saturation curves is the average of 100 repeats of this procedure, explaining the smoothness of the curve even in the cases where the total number of sites is small (e.g. the saturation in functional phosphosites discussed later).

**Figure 3:**
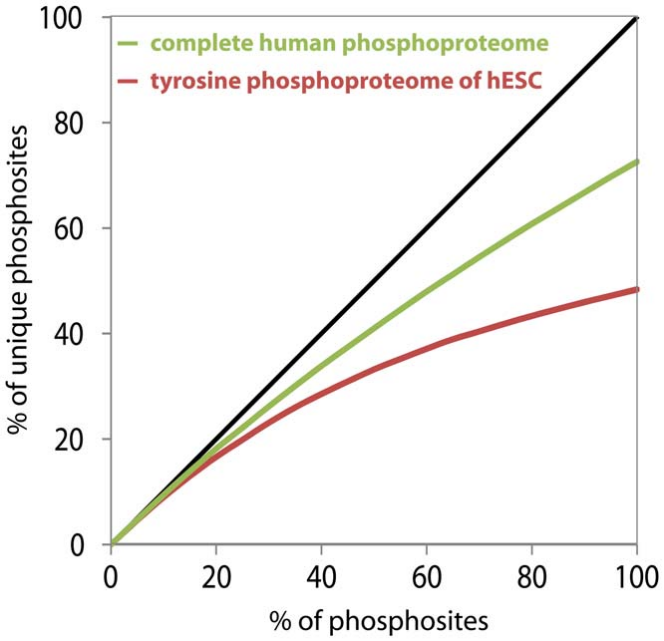
Saturation of the number of observed phosphosites. The tyrosine phosphoproteome of hESC was taken from [12], complete human phosphoproteome data from [13], [14]. The horizontal axis depicts the fraction of phosphosites sampled from the total dataset, the vertical axis depicts the fraction of unique sites. The black line is the diagonal, representing what the curves would look like if there would be no saturation at all (i.e. every site would only be found a single time). Generation and interpretation of the figure is described in more detail in the main text.

This analysis confirms the relatively high saturation of tyrosine phosphorylation in hESC cells (fig 3, red curve). In sharp contrast, the current deposited human phosphoproteome is far from saturation (fig 3, green curve), reflecting the many different techniques used for identifying phosphosites and the myriad of conditions that can be sampled (different cell lines, tissue etc., each potentially with its distinct phosphoproteome). This difference in saturation shows that although combining multiple measurements of a specific biological sample results in a reproducible set of phosphosites, we are still far away from a complete picture of the human phosphoproteome.

**Figure 4:**
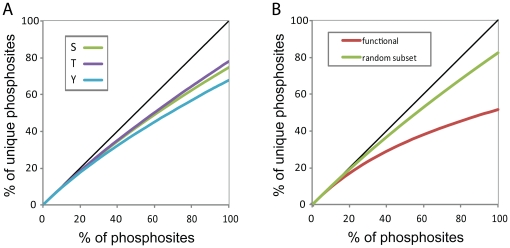
Differences in saturation for different types of phosphosites. The horizontal axis depicts the fraction of phosphosites sampled from the total dataset, the vertical axis depicts the fraction of unique sites. The black line is the diagonal. Figure 3A: phosphosites taken from Phospho.ELM and HPRD [13], [14]. Figure 3B: the functional subset is the functional fraction of Phospho.ELM and HPRD as defined by [17], the random subset is taken from the high-throughput subset of Phospho.ELM and HPRD (sites are considered high-throughput when they have been identified in an experiment identifying 50 or more phosphosites).

The saturation graph follows a curve which could potentially level off or continue to rise as more measurements are added. If this curve would actually saturate, than we can estimate the size of the complete human phosphoproteome by extrapolating from the saturation graph of the 17745 unique phosphorylation sites currently reported by HPRD and phospho.ELM. However several factors compound such an analysis and the choice of function to fit would be arbitrary, and would in fact determine if the graph levels off (and where) or not. This estimation challenge is analogous to rarefaction estimates such as the Chao estimator [15] which is used in ecology to estimate species richness based on the number of observed species. Using the Chao estimator we predicted a total of 57.384 phosphorylation sites in the human phosphoproteome based on the combined HPRD and phospho.ELM (3.2% of the total of 1.783.701 combined S, T and Y residues in the human proteome). However, using a subset of phosphosites as input for the Chao estimator (i.e. jackknifing) consistently results in a lower estimate: using only half of the total number of sites gives an average estimate of 24.402 phosphosites. This unfortunately shows that the assumptions behind the Chao estimator do not hold for the data. One explanation could be false positive phosphosites: a false discovery rate of 1% in a high-throughput MS experiment means that one in every 100 phosphosites identified would appear falsely to be a novel phosphosite, even if a complete human phosphoproteome was already known; as a result the apparent number of unique phosphosites will keep increasing (i.e. curves as presented in figures 3 and 4 will never become horizontal). An alternative and more intriguing explanation is that all S, T or Y residues are phosphorylated to a greater or lesser degree under one or the other condition, an explanation that is analogous to the findings of the ENCODE project [16] that most parts of the human genome are transcribed to a lesser or greater extent. If such pervasive phosphorylation is indeed occurring, we will eventually find all residues in the human proteome to be phosphorylated if we investigate more experimental conditions and keep improving sensitivity of detection methods.

It thus seems that we cannot determine the exact size of the complete human phosphoproteome. Nevertheless the level of saturation of different types of phosphorylation sites reveals interesting biological trends. The targeted sampling of the phosphotyrosine phosphoproteome appears to be more comprehensive than the sampling of the larger and more complex phosphoserine and phosphothreonine proteomes; the final 10% of draws for phosphotyrosines increases the number of unique sites by 5.2% (122 sites), while for phosphoserines and phosphothreonines this is 6.1% (760 sites) and 6.7% (197 sites), respectively (fig 4A). This difference suggests that we are relatively close to a complete picture of the tyrosine phosphoproteome. Combining this with the assumption that the fraction of phosphotyrosines in the phosphoproteome is between 0.1 and 1%, makes it tempting to speculate on the size of the complete human phosphoproteome based on the number of observed phosphotyrosines. However, we have to keep in mind that (part of) the apparent saturation in phosphotyrosines could reflect the more selective nature of phosphotyrosine enrichment strategies (i.e. the majority of phosphotyrosines is derived from analysis using anti-Yp enrichments strategies, which might consistently select for a specific subset of the tyrosine phosphoproteome).

Intriguingly, the saturation also displays a striking difference between non-functional and functional phosphosites: sites that have been experimentally shown to impact phenotypes are much closer to saturation than non-functionally annotated phosphosites (fig 4B). Although the list of functional phosphosites stems from classical molecular biology and biochemistry experiments (compiled by [17] through manual inspection of primary literature), the saturation effect displays itself in high-throughput phosphoproteomics datasets: a total of 41 observations of functional phosphosites in HTP-MS experiments corresponds to 21 unique sites, while for phosphosites of unknown function the same number of observation yields an average of 33.7 unique sites (fig 4B). Randomization trials show that this difference is highly significant (p<0.001) despite the relatively small number of available functionally annotated sites. We have to be careful to attribute this difference to functionality, as it could for example reflect the high abundance of the phosphosite. Nevertheless, the high saturation of functional phosphosites suggests that we are in fact closer to a complete “biologically relevant” human phosphoproteome than the saturation curve of all observed phosphosites implies.

### Enrichment bias

Enrichment for phosphopeptides is a crucial step in a high-throughput MS-based phosphoproteomics experiment because of their low abundance and stoichiometry. The exact method of enrichment has a significant impact on the phosphoproteome measured: different techniques –based on antibodies, TiO2 or IMAC using various metal ions- are thought to give overlapping but distinct segments of a phosphoproteomes [10], [18]. This effect is likely a prominent factor in the saturation curves discussed above, but its exact nature and the size of its contribution cannot be readily determined from such curves. Furthermore, the applied proteolytic enzymes can have an effect on the coverage of the phosphoproteome. The negative phosphate group typically lowers the digestion efficiency of trypsin that is typically utilized in phosphoproteomics experiments. Therefore, alternative enzymes may cover complementary stretches of phosphoproteins [11].

We analyzed the nature of impact of enrichment strategy (the step indicated with a * in fig 1) by comparing the amino acid composition of phosphopeptides found using the targeted anti-Yp enrichment of phosphotyrosine peptides *versus* the composition of phosphotyrosine-containing peptides obtained from more general phosphopeptide enrichment strategies. The amino acid composition is significantly different for phosphopeptides enriched using anti-Yp [19] and phosphopeptides enriched using the broader TiO_2_ affinity enrichment of phosphopeptides (Table 1). For example, the prevalence of tyrosine residues is more than three times as high in the peptides detected by using anti-Yp when compared to those detected in TiO_2_ affinity enriched fractions [20], possibly influenced by the presence of multiple phosphorylated tyrosines in the same peptide. Conversely peptides purified by the TiO_2_ enrichment strategy contain many more negatively charged amino acids such as glutamate or aspartate, not surprisingly as TiO_2_ is known to bind also acidic peptides [10].

**Table 1:** Effect of enrichment strategy on amino acid composition.

	Prevalence (%)*	
residue	TiO_2_	anti-Yp
D	13.0	7.4
E	13.6	8.1
Y	1.2	4.2
S	13.6	9.4
Q	2.6	4.5
K	2.6	5.5

The amino acid composition of regions surrounding phosphorylated tyrosines (5 residues on each side) found in experiments using anti-Yp enrichment (data from [8], [21], [26]) was compared to the composition of areas surrounding tyrosines found using TiO_2_ affinity (data from [22]). Only residues with a significantly different prevalence are shown (p<0.01).

*the amino acid prevalence does not include the central tyrosine itself.

The impact of enrichment strategies on the overlap between different experiments cannot be quantified by directly comparing two datasets, as the actual number of phosphosites in the samples is not known and likely much larger (we do not know how many of the phosphosites present in the sample were not measured, i.e. false negatives): lack of overlap is a combination of both the incomprehensive nature of the experiments and the impact of enrichment strategies. We try to disentangle these two factors through a common reference approach, in which differences in relative overlap between two datasets to a common benchmark reflect the bias between these two datasets. As a reference we took the tyrosine phosphoproteome described in [21] , which was generated through anti-tyrosine enrichment (1992 phosphotyrosines from lung cancer cell lines). To this reference we compared a dataset also generated using anti-Yp enrichment ([8], 798 phosphotyrosines in HeLa cells) and the phosphotyrosine component of a dataset using TiO_2_ enrichment ([22], 82 phosphotyrosines from HeLa cells). The fraction of phosphosites present in the large reference dataset is quite different for the anti-Yp enriched dataset and phosphopeptides enriched using TiO_2_ affinity enrichment. From the anti-Yp experiment 38% of the sites was also found in the reference, while for the TiO_2_ experiment only 16% was found. These ratios allows us to estimate what the overlap would have been if two experiments would have used the same techniques: when comparing TiO_2_-derived datasets to anti-Yp derived we could correct for bias introduced by enrichment strategy by multiplying the observed overlap by 38/16 = 2.4.

The number of phosphotyrosines in the TiO_2_ dataset used in this comparison is relatively small; this is a direct result of the low abundance of phosphotyrosine compared to phosphoserines and phosphothreonines, combined with an enrichment strategy that does not specifically target phosphotyrosines. Still, procedures like the one given above enable us thus to obtain a concrete estimate of the size and nature of the enrichment bias, improving the usability of the ever-increasing amount of phosphoproteomics data available in the public domain for large-scale comparative analysis into the dynamics and evolution of the phosphoproteome.

### Inter-species comparative phosphoproteomics

Inter-species comparison of phosphoproteomes is confounded by enrichment bias described above, as datasets from different species were most likely obtained using different growth and sampling strategies. Despite these hurdles we and others have identified both a functional and an evolutionary signal in the overlap between phosphoproteomes from different species of eukaryotes [2], [5], [6]. Still, the absolute overlap is very low. In light of the issues described above, it is likely that this overlap not only reflects the speed at which phosphoregulation evolves, but in part also reflects differences in experimental techniques as well as sampling strategies and conditions.

Hence we test the size of the overlap using the same experimental pipeline to compare phosphoproteomes from widely diverged species. Specifically we measured the tyrosine phosphoproteome of *C. elegans* and determined the overlap with the tyrosine phosphoproteome of human HeLa cells as presented in [8]. We minimized the impact of differences in experimental procedures on the observed overlap by sticking as close to protocols used for the measurement of the HeLa cell phosphoproteome (more details in the methods section). We identified 226 unique phosphorylation sites in *C. elegans* in a total of 131 different proteins (table S1). For 9 of these sites (4%) an orthologous phosphorylation event was present in the HeLa dataset. Kinases are strongly overrepresented in the overlap: of the 9 conserved sites, 7 are located in a kinase, while for the total 268 sites this is 25 (p<1e-06, Fisher's exact test), showing that the phosphorylation machinery itself is not only overrepresented in the phosphoproteome compared to the complete proteome of *C. elegans* (fig S1), but especially so in the conserved fraction of the phosphoproteome.

The overlap of 4% between the *C. elegans* and human datasets is similar to the overlap between yeast phosphoproteomes as published recently by [2] who also employed comparable experimental strategies. Although 4% is a low percentage, it still represents a three-fold improvement over the overlap between datasets from different species ([22] and [10]) analyzed in a previous study: if we determine the overlap between these two old datasets with the methods we used for the *C. elegans* and human presented above, we find an overlap of only 1% (23 of the 2080 phosphosites in the old fly dataset have an orthologous site in the old human dataset). Moreover, we need to keep in mind that we can reduce the biases by using comparable experimental setup, but we are still hindered by the non comprehensive nature of the HTP MS experiment. Nevertheless the fourfold increase illustrates the impact of technique on observed overlap and shows that inter-species comparative phosphoproteomics can be fruitful as long as datasets are generated using similar techniques. Barring that possibility we conclude that differences in techniques should be taken into account when interpreting comparative results, which means that low overlap between species is not automatically completely attributed to evolutionary events when technical explanations also play a large role.

### Summarizing conclusion

We have shown how saturation graphs can be used to visualize the relation between the number of technical replicates of an experiment and the number of unique phosphosites found, which in turn can be of assistance in deciding whether or not to do additional replicates. Combining multiple measurements of a specific biological sample results in a reproducible set of phosphosites, but we are still far away from a complete picture of the human phosphoproteome. Another factor confounding the comparative analysis of phosphoproteomics datasets are the biases introduced at different points in the high-throughput phosphoproteomics pipeline. We have presented a common-reference based strategy that quantifies the bias introduced by different enrichment strategies, and show that using the same experimental pipeline greatly increases the power of inter-species comparative phosphoproteomics.

We expect that both experiments specifically targeted at the analysis of dynamics and evolution of the phosphoproteome as well as the comparative analysis of the ever-increasing amount of publically available phosphoproteomics data will be crucial in elucidating the function of specific phosphorylation events and the interplay between phosphorylation and the other dynamic biological networks.

## Materials and Methods

### Datasets

The specific phosphoproteomics datasets used in the analysis are mentioned in the main text and figure legends. Phosphosites were mapped to reference proteomes to allow the combination and comparative analysis of different datasets, resulting in small differences in the number of sites used in our analysis and the number of sites reported in their original publication. Data handling was done using ad hoc Python scripts.

### Inter-species comparative analysis

A phosphorylation event in a query species was considered to be conserved in a target species when a sequence alignment of the query protein with an orthologous protein from the target species exactly aligns the phosphorylated site in the query protein with a phosphorylated residue in the target sequence. Orthologous proteins were identified using Inparanoid [23], sequence alignments were made using Muscle [24].

### Amino acid prevalence

Amino acid prevalence was measured in the 10 residues flanking a phosphosite (5 N-terminal and 5 C-terminal). The phosphorylated residue itself was not included. The significance of a difference in prevalence was determined though randomization trials (10000 runs), in which sites were randomly assigned to a purification strategy (the total number of sites in each set was kept constant). P-values were corrected for multiple testing using Bonferroni correction.

### Analysis of the C. elegans phosphoproteome

The Bristol N2 strain was used as standard wild-type strain. CB3241 was obtained from the Caenorhabditis Genetics Center. Standard *C. elegans* culturing methods were used. Animals were staged by hatching eggs overnight in M9 medium. Staged L1 larvae were cultured for 3 hours at the permissive temperature of 15°C and subsequently rinsed of the culturing plates and incubated at the restrictive temperature of 25°C. Animals were vacuum dried in a speedvac and the pellet was resuspended in 8 M urea and sonicated (5×30″ at maximum intensity) in a sonication bath. The supernatant was used for trypsin digestion. Proteins were reduced with 1 mM DTT and alkylated with 2 mM iodoacetamide. Prior to digestion the samples were diluted to a final concentration of 2 M urea in a final concentration of 50 mM ammonium bicarbonate. Proteins were digested overnight at 37°C using trypsin (final concentration: 1.0 ug/ml).

### Sample preparation and immunoprecipitation

Labeled peptides were desalted, dried down and re-dissolved in IP buffer (50 mM Tris; pH 7.4, 150 mM NaCl, 1% NOG). Prior to immunoprecipitation, pY99 agarose beads (Santa Cruz Biotechnology Inc., CA) were washed in IP buffer. The peptide mixture was added to the pY99 agarose beads and incubation was performed overnight at 4°C. Beads were washed several times with IP buffer and milliQ. Peptides were eluted with 0.15% Trifluoroacetic acid for 20′ at RT. Eluted peptides were desalted and concentrated on STAGE-tips.

### On-line nanoflow liquid chromatography

Nanoflow LC-MS/MS was performed using an Agilent 1100 HPLC system (Agilent Technologies, Waldbronn, Germany), coupled to an LTQ-Orbitrap mass spectrometer (Thermo Electron, Bremen, Germany). Dried fractions were reconstituted in 15 µL 0.1 M acetic acid and delivered to a trap column (Aqua^tm^ C18, 5 µm, Phenomenex, Torrance, CA, USA); 20 mm×100 µm ID, packed in-house) at 5 µL/min in 100% solvent A (0.1 M acetic acid in water). Peptides were subsequently transferred to an analytical column (ReproSil-Pur C18-AQ, 3 µm, Dr. Maisch GmbH, Ammerbuch, Germany; 40cm×50 µm ID, packed in-house) at ∼100 nL/min in a 3 hr gradient from 0 to 40% solvent B (0.1 M acetic acid in 8/2 (v/v) acetonitrile/water). The eluent was sprayed via distal coated emitter tips (New Objective), butt-connected to the analytical column. The mass spectrometer was operated in data dependent mode, automatically switching between MS and MS/MS. Full scan MS spectra (from m/z 300–1500) were acquired in the Orbitrap with a resolution of 60,000 at m/z 400 after accumulation to target value of 500,000. The three most intense ions at a threshold above 5000 were selected for collision-induced fragmentation in the linear ion trap at normalized collision energy of 35% after accumulation to a target value of 10,000.

### MS data analysis

All MS2 spectra were converted to single .DTA files using Bioworks 3.3. Runs were searched using an in-house licensed MASCOT search engine (Mascot (version 2.1.0) software platform (Matrix Science, London, UK)) against the *Caenorhabditis elegans* predicted proteome with carbamidomethyl cysteine as a fixed modification. Oxidized methionines and phosphorylation of tyrosine were set as variable modifications. Trypsin was specified as the proteolytic enzyme and up to two missed cleavages were allowed. The mass tolerance of the precursor ion was set to 5 ppm and that of fragment ions was set to 0.6 Da. Individual MS/MS spectra from phosphopeptides were accepted for a Mascot score ≥20.

## Supporting Information

Figure S1 **Overrepresentation of GOslim terms** [**25**] **of phosphoproteins relative to the** ***C. elegans*** **proteome.** Overrepresentation is expressed as fold increase: 2log(fraction_phosphoproteome) - 2log(fraction_proteome). The numbers in the bars are the number of phosphoproteins associated with the GOslim term, the numbers in italics to the right of every bar is the significance (Fisher exact test, p-value after Benjamini & Hochberg multiple testing correction).
(TIF)

Table S1 **Phosphorylation sites in** ***C. elegans*** **.**
(XLS)
