# Supplementary figures and images for: Evaluating Experimental Bias and Completeness in Comparative Phosphoproteomics Analysis
Source: PLoS One. 2011 Aug 10;6(8):e23276. doi: 10.1371/journal.pone.0023276 (PMC3154393; doi:10.1371/journal.pone.0023276)

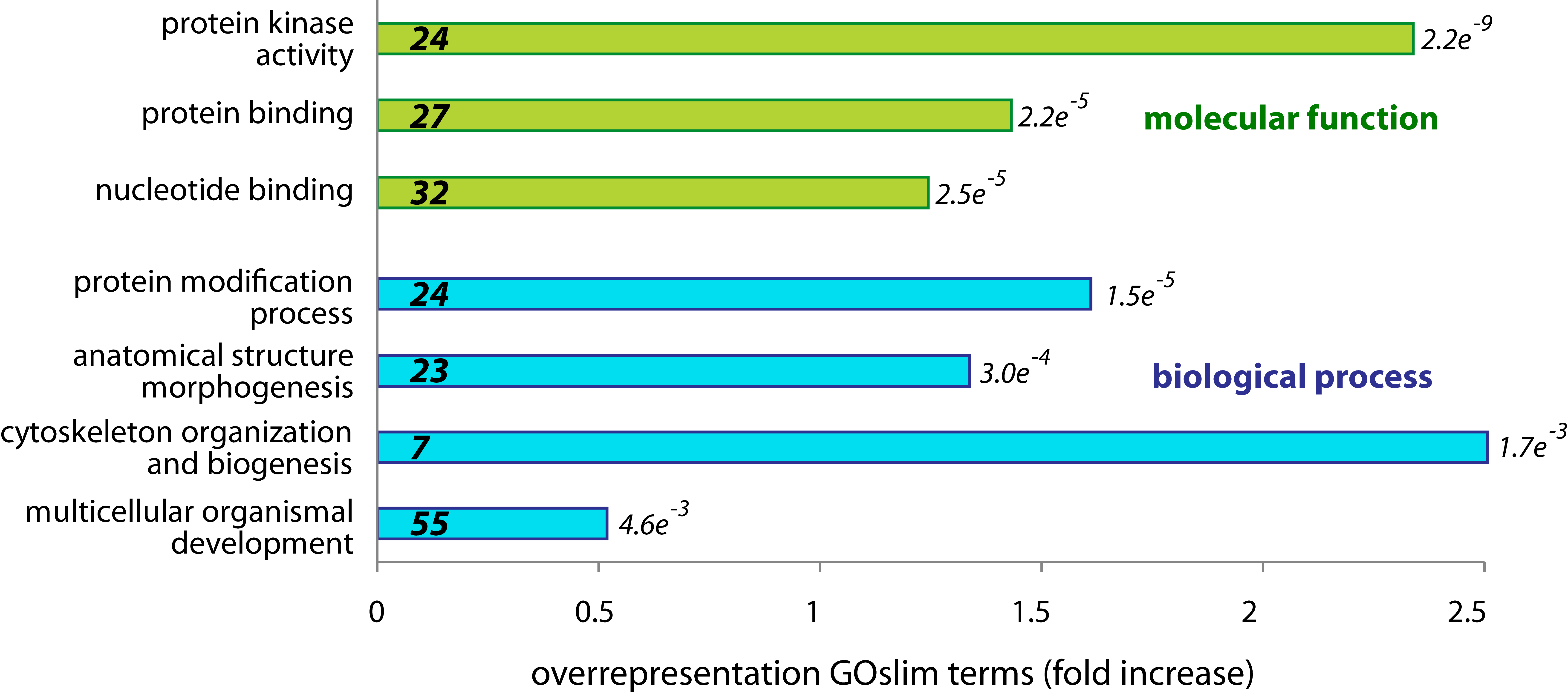

Supplement: Figure S1 — Overrepresentation of GOslim terms [25] of phosphoproteins relative to the C. elegans proteome. Overrepresentation is expressed as fold increase: 2log(fraction_phosphoproteome) - 2log(fraction_proteome). The numbers in the bars are the number of phosphoproteins associated with the GOslim term, the numbers in italics to the right of every bar is the significance (Fisher exact test, p-value after Benjamini & Hochberg multiple testing correction). (TIF) [file pone.0023276.s001.tif]
